# Contralateral C7 transfer to axillary and median nerves in rats with total brachial plexus avulsion

**DOI:** 10.1186/s12891-020-03209-1

**Published:** 2020-03-28

**Authors:** Yuzhou Liu, Feng Xiao, Yongqing Zhuang, Jie Lao

**Affiliations:** 1 Department of Hand Surgery, Huashan Hospital, Fudan University, 12 Wulumuqi Zhong Road, Jing An District, Shanghai, 200040 China; 2 Key Laboratory of Hand Reconstruction, Ministry of Health, Shanghai, China; 3 Shanghai Key Laboratory of Peripheral Nerve and Microsurgery, Shanghai, China; 4 Hand and Microvascular Surgery Department, Shenzhen People’s Hospital, The 14th Floor of the Surgery Building, East Gate Road 1017Luohu District, Shenzhen, 518020 Guangdong Province China

**Keywords:** Brachial plexus avulsion, Nerve transfer, Contralateral C7, Axillary nerve, Median nerve

## Abstract

**Background:**

Contralateral cervical 7 nerve (cC7) was used to repair two recipient nerves simultaneously for patients with total brachial plexus avulsion (TBPA).

**Objective:**

To evaluate the effect of cC7 transfer to axillary and median nerves in rats with TBPA.

**Methods:**

Eighty S-D rats were divided into 4 groups randomly on average. Group A: cC7-median nerve, Group B: cC7-axillary nerve, Group C: cC7-median and axillary nerves, Group D: TBPA without repair. The evaluation tools included behavioral tests, electromyogram (EMG), measurement of cross-sectional area of muscle fiber, nerve fiber count and gene expression assay.

**Results:**

The effective rates of EMG were 90 and 70% in Flexor Carpi Radialis (FCR) in Group A and C, while 70 and 60% in deltoid (DEL) in Group B and C, respectively. In behavioral test, the differences of effective rates between groups were not significant. The mean cross-sectional area of FCR in Group A or C was significantly larger than that in Group D. Either the number of median or axillary nerve fibers in Group A, B or C was statistically more than that in Group D. No matter for FCR or DEL, there were no significant differences in the ratios of relative expression of Muscle Atrophy F-box(MAFBOX)and Muscle RING Finger 1(MURF1)among these groups.

**Conclusion:**

Compared with cC7 transfer to median nerve, cC7 transfer to both median and axillary nerves did not affect median nerve recovery. The deltoid muscle also could be restored. The recovery proportion of axillary nerve was less than that of median nerve.

## Background

In recent years, nerve transfer has been the main treatment of total brachial plexus avulsion (TBPA). The functional improvements are mainly embodied in shoulder abduction and elbow flexion. However, the recoveries of shoulder external rotation and intrinsic function are not satisfying [17, 22]. According to the anatomy, the contraction of muscle fibers in the posterior part of deltoid muscle (DEL) innervated by the axillary nerve results in shoulder external rotation. Therefore, axillary nerve recovery could improve shoulder external rotation theoretically.

Contralateral cervical 7 nerve (cC7) transfer is usually used to repair median nerve at the affected side. It’s effective in restoring partial sensation of hand,wrist and finger flexion without permanent sensory and motor damages to the healthy side [3, 9]. Because the number of myelinated nerve fibers of cC7 root is much more than that of any recipient nerve including radial nerve, median nerve or musculocutaneous nerve, some scholars have tried to repair two recipient nerves simultaneously by cC7 transfer [7, 24, 25, 27]. They have found that both of the two recipient nerves have achieved recoveries. However, there was no report on cC7 transfer to median and axillary nerves simultaneously. Therefore, we carried out an animal experimental research to study the effect of cC7 transfer to both median and axillary nerves. The objective of the research was to establish an animal experimental foundation for the further clinical application.

## Methods

### Animals and preparation before surgery

Male Sprague-Dawley(S-D)rats (*n* = 80; weight, 200–250 g; age, 8 weeks) were kept in an environment with the temperature of 20 °C and humidity of 50%. All the rats were supplied by the company (Shanghai Slake Laboratory Animals Company, Shanghai, China). The rats were maintained on a 12/12-h light/dark cycle and allowed free access to food and water.

Eighty rats were divided into 4 groups randomly on average as follows:

- Group A: Rats with TBPA and cC7 transfer to median nerve (cC7 --- median nerve).
- Group B: Rats with TBPA and cC7 transfer to axillary nerve (cC7 --- axillary nerve).
- Group C: Rats with TBPA and cC7 transfer to both median and axillary nerves (cC7 --- median and axillary nerves).
- Group D: Rats with TBPA without repair.

The right side of each rat was selected as the injure side in all groups. Intraperitoneal injection of 1% pentobarbital sodium (1 ml/100 g body weight; Shanghai Reagent Company, Shanghai, China) was used before operation. After anesthesia, the rats were fixed and disinfected in the supine position.

### Surgical techniques

#### TBPA rat model

A right supraclavicular incision was made. Part of sternocleidomastoid and anterior scalene muscles were cut off. C5 and C6 nerve roots were identified. Then the clavicle was pulled down to explore C7, C8 and T1. After the right brachial plexus was completely exposed, the C5-T1 nerve roots were pulled out until avulsion at the foramen levels in Group A, B, C and D.

#### The first stage of cC7 transfer

CC7 nerve transfers were performed after TBPA in Group A, B, and C. A longitudinal incision was made along the ulnar side of the right upper limb. The ulnar nerve was exposed from the wrist to the axilla. Then the ulnar nerve was cut off at the wrist and separated from the wrist to the axilla. The superior ulnar collateral artery was reserved. A transverse incision was made on the left supraclavicular fossa. The cervical 7 nerve root was explored and separated from the intervertebral foramen. 2% lidocaine (Shanghai Reagent Company, Shanghai, China) was used for nerve block before the whole cC7 root was cut. The distal end of the right ulnar nerve was transferred to the left supraclavicular fossa through a subcutaneous tunnel. It was sutured to the left whole cervical 7 nerve root.

#### The second stage of cC7 transfer

**Fig. 1:**
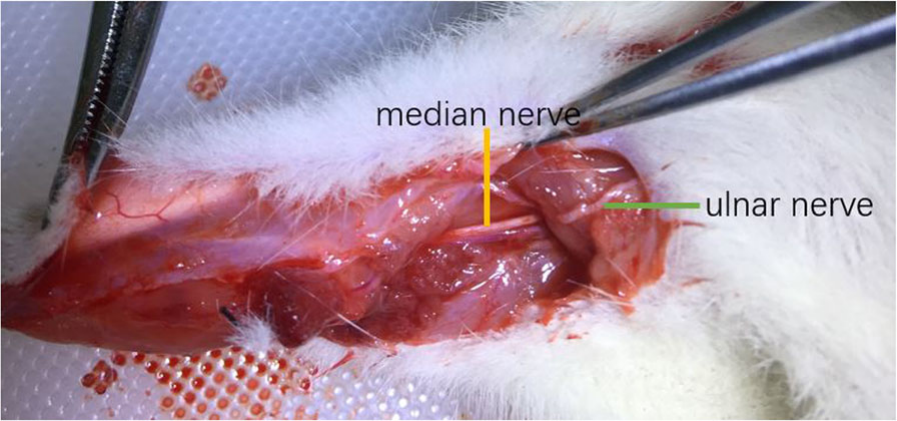
cC7 transfer to median nerve: The ulnar nerve and median nerve were identified at the turning point of ulnar nerve

Eight weeks after the first stage of cC7 transfer, the rats in Group A, B and C underwent the second stage of cC7 transfer. The procedure in the second stage of cC7 transfer in each group was as follows:

Group A (cC7 --- median nerve): A right longitudinal incision was made on the inner side of the upper arm. The ulnar and median nerves were identified near the retracing point of the ulnar nerve. (Fig. 1) Both of them were cut off. The proximal end of the ulnar nerve was sutured to the distal end of the median nerve without tension.

**Fig. 2:**
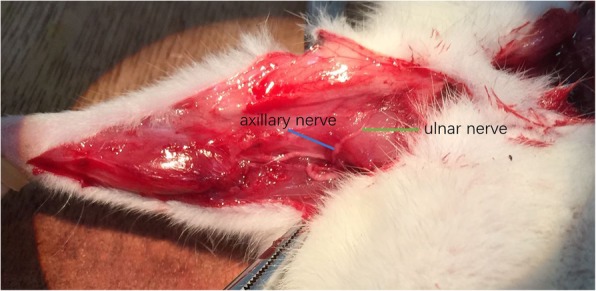
cC7 transfer to axillary nerve: The proximal end of ulnar nerve was sutured to the distal end of the whole axillary nerve without tension

Group B (cC7 --- axillary nerve): A Z shape incision was made in the right axilla. The axillary nerve was found at the quadrilateral foramen. The ulnar nerve was exposed at the retracing point. The axillary nerve was separated and cut off. The ulnar nerve was cut in the axilla. (Fig. 2) Then the proximal end of ulnar nerve was sutured to the distal end of the whole axillary nerve without tension.

**Fig. 3:**
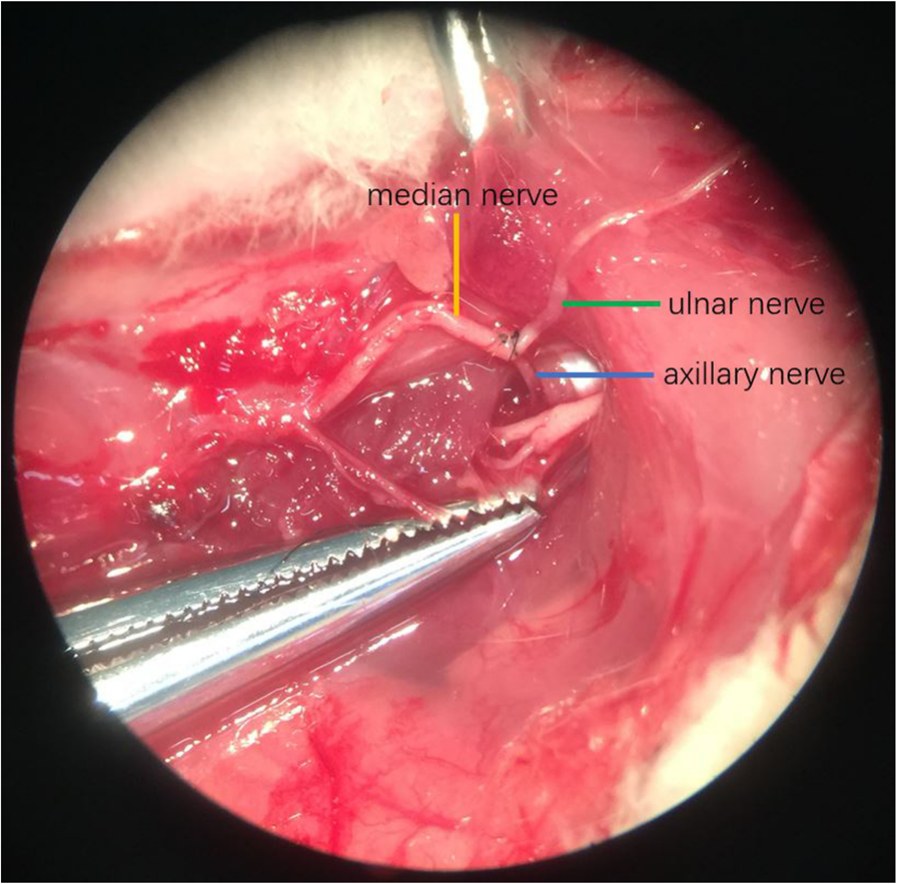
cC7 transfer to median and axillary nerves: Axillary nerve and median nerve were separated to enough length for suturing to the ulnar nerve without tension

Group C (cC7 --- median and axillary nerves): A Z shape incision was made to expose the ulnar nerve, median nerve and axillary nerve in the right axilla. The axillary nerve was cut off at the quadrilateral foramen. The ulnar and median nerves were separated to enough length for suture in the axilla. Then they were cut off. The proximal end of ulnar nerve was sutured to the distal ends of the axillary and median nerves without tension. (Fig. 3).

In the experiment, the nerves were sutured with 11–0 nylon (Ethicon, Johnson & Johnson, New Brunswick, New Jersey) under 10 times microscope.

### Evaluation methods

All the examinations and evaluations were carried out 12 weeks after the second stage of cC7 transfer. First, the behavioral tests were made in the rats. Then we used electromyogram (EMG) examinations (Neuromatic 2000 M electrophysiological apparatus, Dantec, Les Ulis, France) to evaluate nerve regeneration. The rats, were anesthetized by 1% pentobarbital sodium intraperitoneal injection before EMG examinations. After EMG examinations, the median and axillary nerves were biopsied for nerve fibers count. Flexor Carpi Radialis (FCR) and deltoid (DEL) were biopsied for calculating the mean cross-sectional areas of muscle fibers. Gene expressions related to muscle atrophy were assayed by RT-qPCR (Agilent Technologies, Inc., Santa Clara, CA, USA).

#### Behavioral test

In Group A, the grasping test was performed. The rat was put on a wire mesh. Under normal circumstances, its paw would do the grasp action to catch the wire. We divided the results into three categories: M0, M1, M2. M0: no digit flexion was observed. M1: digit flexion was observed without grasping. M2: grasping was observed. We defined M1 and M2 as effective recoveries for digit flexion.

In Group B, water spray test was performed. The rat’s posterior skull was continuously sprayed with a syringe. The rat would do shoulder abduction and external rotation to touch the wet position if the axillary nerve recovered. We recorded the number of rats with shoulder abduction and external rotation. Then we calculated the effective rate.

The grasping test and water spray test were carried out in Group C.

#### EMG examination

The rats were anesthetized with sodium pentobarbital injected intraperitoneally. A transverse incision was made on the left supraclavicular fossa to expose the bridging ulnar nerve. A stimulating electrode was put on the bridging ulnar nerve. A recording electrode was inserted into the target muscle. A current of 1 Hz, 0.5 mA was used for stimulating the nerve.

- Group A: the recording electrode was inserted into the FCR.
- Group B: the recording electrode was inserted into the DEL.
- Group C: the recording electrodes were inserted into FCR and DEL.

The emergence of compound muscle action potential (CMAP) was regarded as an effective recovery for motor function.

#### Cross-sectional area of the muscle fiber

FCR and DEL were biopsied from the affected side in Group A and B, respectively. Both of the two muscles were obtained in Group C and D. All the muscles were fixed in 10% paraformaldehyde (Shanghai Reagent Company, Shanghai, China). Then they were fixed in paraffin (Shanghai Reagent Company, Shanghai, China). The muscles were stained with hematoxylin and eosin(HE)(Shanghai Reagent Company, Shanghai, China). The mean cross-sectional area of the muscle fiber was measured and calculated by Image J system (National Institutes of Health, USA).

#### Nerve fiber count

Median and axillary nerves were biopsied from the affected side in Group A and B, respectively. Both of the two nerves were obtained in Group C and D. All nerve sections were taken near their entry points into the target muscles. The samples were fixed in 25% glutaraldehyde (Shanghai Reagent Company, Shanghai, China) and embedded with paraffin for slices. Then they were stained with 5% toluidine blue (Shanghai Reagent Company, Shanghai, China). The average number of nerve fibers in unit area was measured and calculated by the Image J system.

#### Gene expressions related to skeletal muscle atrophy

A previous study was reported [5] that Muscle RING Finger 1(MuRF1)and Muscle Atrophy F-box (MAFbx)were the genes related to skeletal muscle atrophy. They were positively correlated with muscle atrophy. In Group A and B, FCR and DEL were biopsied respectively. Both of the two muscles were biopsied in Group C and D. Muscle tissue samples were dissolved in TRIzol™ (1600 Faraday Ave, Carlsbad CA92008 USA). Then DNA was separated, precipitated and extracted from the samples. The purity of the DNA was determined. Expression of each gene was assayed by RT-qPCR (Agilent Technologies, Inc., Santa Clara, CA, USA). The relative expression levels of the two genes were determined using 2^-ΔΔCt^ method.

After the completion of the biopsy, each group was euthanized using CO2. The flow rate for CO2 euthanasia displaced 20% of the chamber volume/min according to the 2013 edition of the American Veterinary Medical Association Guidelines for the Euthanasia of Animals.

### Date analysis

Comparisons of the effective rates among different groups were performed using Fisher’s exact test in behavioral test and EMG. One-way ANOVA (analysis of variance) was used in the aspects of cross-sectional area of the muscle fiber, nerve fiber count and gene expressions. The *p*-values were two-tailed and p-values < 0.05 were considered significant. All analyses were performed using Statistical Package for Social Sciences (SPSS, Chicago, IL, USA) 24.0 software.

## Results

Two rats died after operations in Group C. We supplemented two rats into Group C and did the operations under the same conditions. Finally, no wound infection was found in any surviving rat.

### Behavior test

According to the grasping test, 14 rats (70%) and 12 rats (60%) achieved effective digit flexion recoveries in Group A and C respectively, while there was no significant difference between the two groups. We observed 12 rats and 8 rats had shoulder abduction and external rotation in Group B and C, respectively. The effective rate in Group B (60%) was larger than that in Group C (40%). However, there was no statistical difference between the two groups. In Group C, the effective rate of digit flexion recovery (60%) was larger than that of shoulder abduction and external rotation recovery (40%), although there was no significant difference. The effective rate of digit flexion recovery in Group A (70%) was larger than that of shoulder abduction and external rotation recovery in Group B (60%). (Table 1).

**Table 1:** The results of behavioral tests and EMG in Group A, B, C

Group	Digit flexion		Shoulder abduction and external rotation		Effective Rate (%)	EMG (FCR)		EMG (DEL)		Effective Rate (%)
	(+)	(−)	(+)	(−)		(+)	(−)	(+)	(−)	
A	14	6	/	/	70%	18	2	/	/	90%
B	/	/	12	8	60%	/	/	14	6	70%
C (FCR)	12	8	/	/	60%	14	6	/	/	70%
C (DEL)	/	/	8	12	40%	/	/	12	8	60%

### EMG examination

Table 1 showed EMG results in FCR and DEL. CMAP in FCR could be recorded in 18 rats (90%) and 14 rats (70%) in Group A and C, respectively. There was no significant difference between the two groups. 14 rats (70%) and 12 rats (60%) had CMAP in DEL in Group B and C, respectively. No statistical difference existed between the two groups. In Group C, the effective rate in FCR (70%) was larger than that in DEL (60%) without a significant difference. In addition, the effective rate in FCR in Group A (90%) was larger than that in DEL in Group B (70%), although there was no statistical difference.

### Cross-sectional area of the muscle fiber

**Fig. 4:**
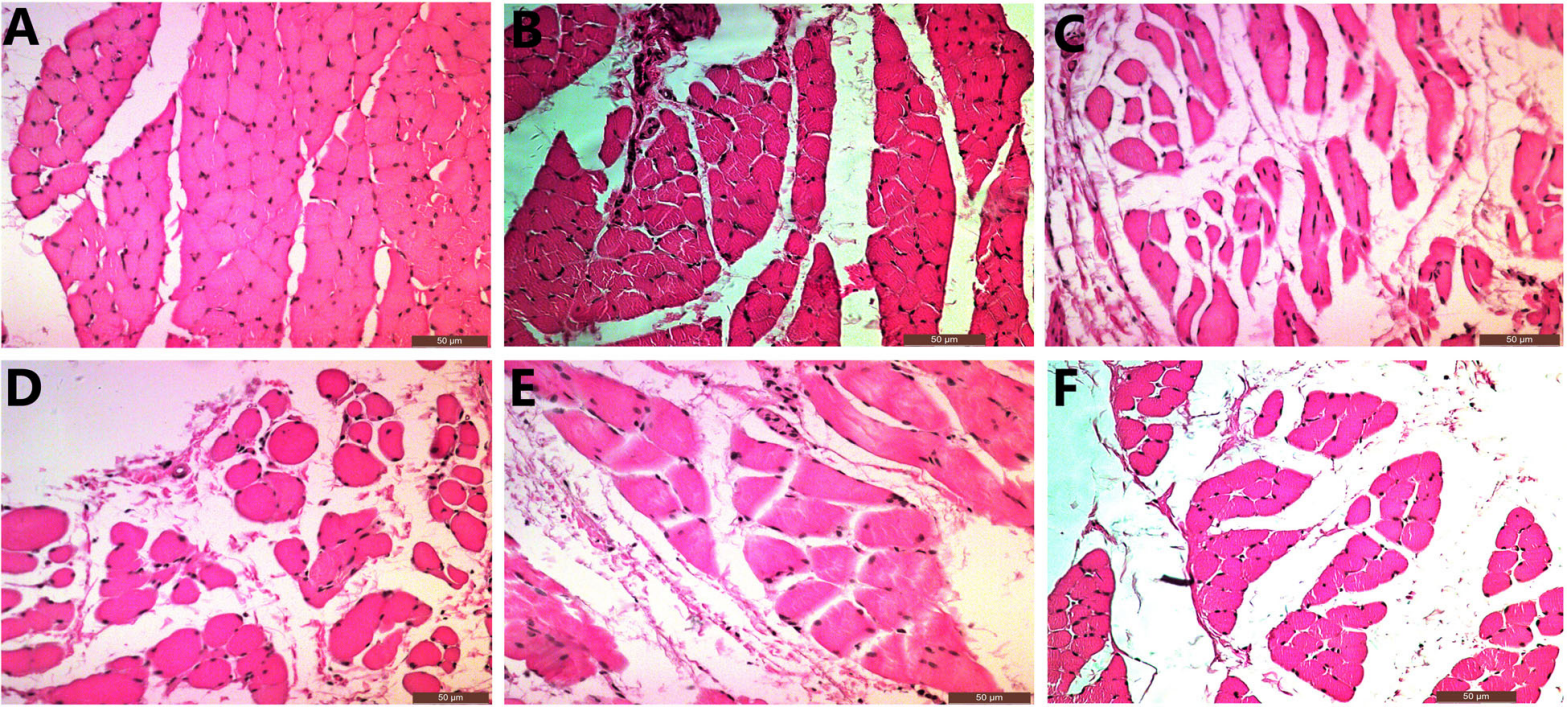
**a** The cross-section of FCR muscle fibers in Group A. **b** The cross-section of FCR muscle fibers in Group C. **c** The cross-section of FCR muscle fibers in Group D. **d** The cross-section of DEL muscle fibers in Group B. **e** The cross-section of DEL muscle fibers in Group C. **f** The cross-section of DEL muscle fibers in Group D

**Fig. 5:**
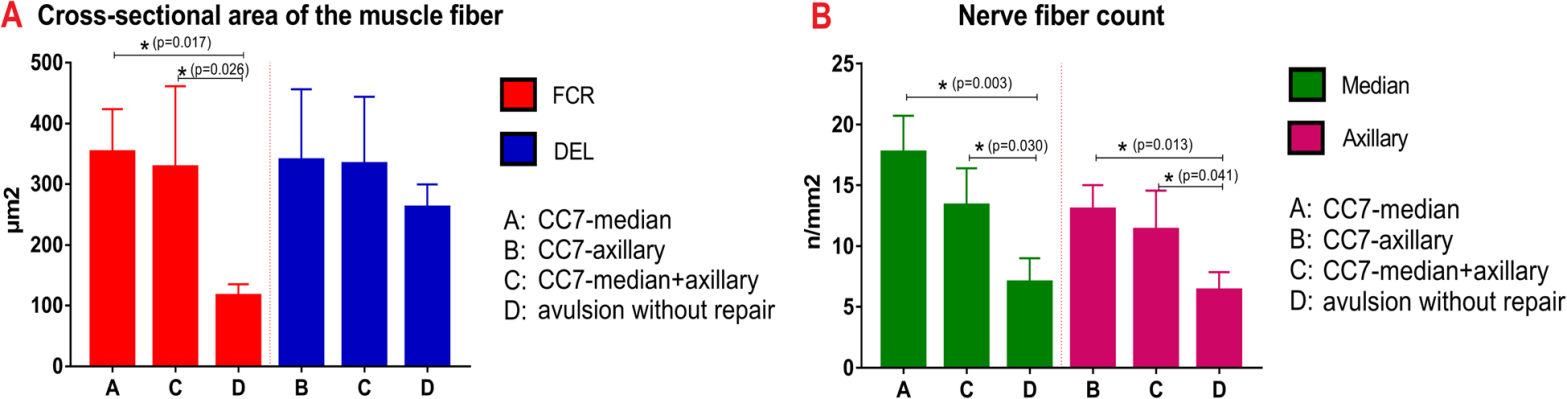
**a** The mean cross-sectional areas of FCR and DEL muscle fibers in different groups. **b** The nerve fiber counts of median and axillary nerves in different groups

We evaluated the mean cross-sectional areas of FCR and DEL muscle fibers to represent the recoveries of median and axillary nerves respectively. (Fig. 4) The average cross-sectional areas of FCR were 352.44 ± 71.33 μm^2^ in Group A and 327.71 ± 134.11 μm^2^ in Group C. There was no statistical difference between the two groups. Either the cross-sectional area in Group A or C was significantly larger than that in Group D (115.65 ± 19.46 μm^2^). The mean cross-sectional areas of DEL were 339.09 ± 117.69 μm^2^, 332.75 ± 111.54 μm^2^ and 261.61 ± 37.78 μm^2^ in Group B, C, D, respectively. There were no statistical differences among the three groups. (Fig. 5a).

### Nerve fiber count

**Fig. 6:**
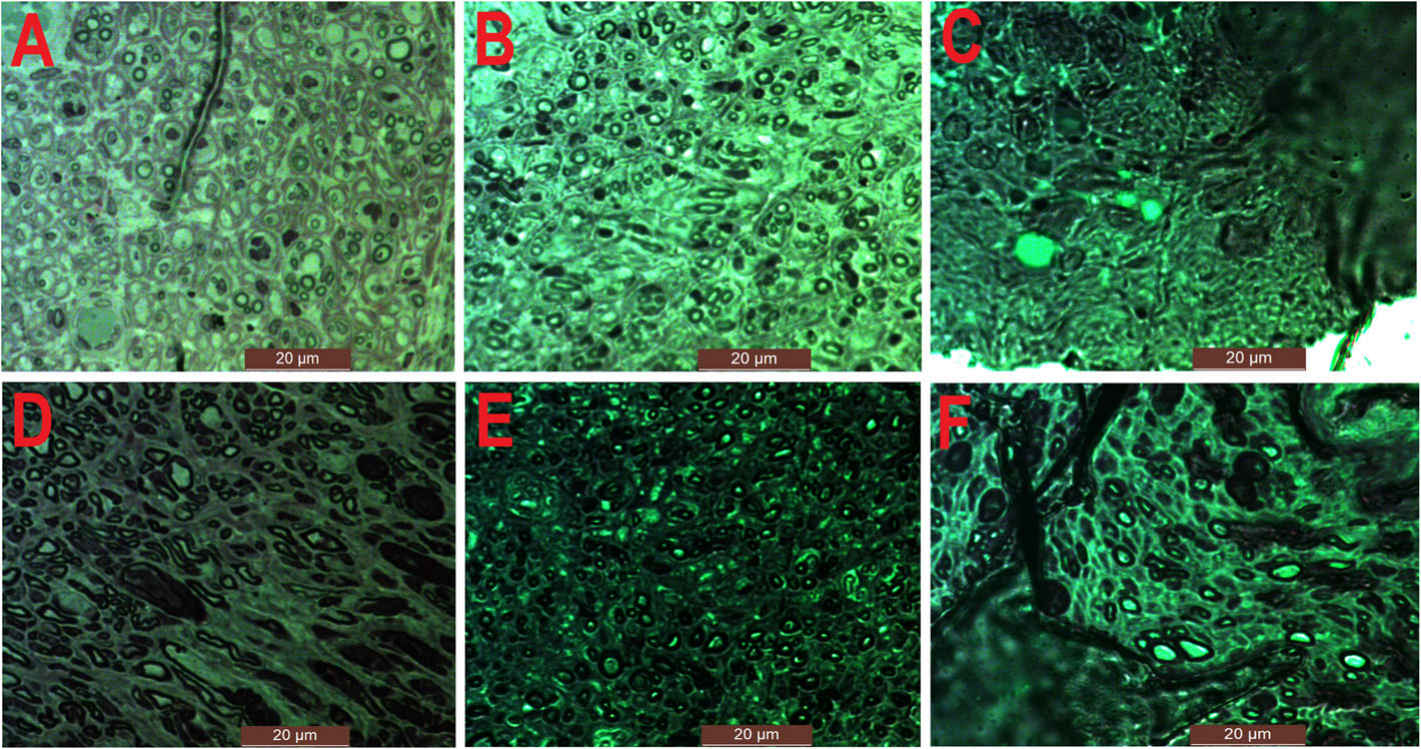
**a** The nerve fibers of median nerve in Group A. **b** The nerve fibers of median nerve in Group C. **c** The nerve fibers of median nerve in Group D. **d** The nerve fibers of axillary nerve in Group B. **e** The nerve fibers of axillary nerve in Group C. **f** The nerve fibers of axillary nerve in Group D

Figure 5b showed the differences in the number of nerve fibers in different groups. The numbers of median nerve fibers were 17.67 ± 3.06/mm^2^ in Group A and 13.33 ± 3.06/mm^2^ in Group C. There was no significant difference in the number of median nerve fibers between the two groups. However, either the number of median nerve fibers in Group A or C was significantly more than that in Group D (7.00 ± 2.00/mm^2^). The numbers of axillary nerve fibers were 13.00 ± 2.00/mm^2^, 11.33 ± 3.21/mm^2^ and 6.33 ± 1.53/mm^2^ in Group B, C and D, respectively. Either the number of axillary nerve fibers in Group B or C was significantly more than that in Group D. There was no statistical difference in the number of axillary nerve fibers between Group B and C. In Group C, the number of median nerve fibers (13.33 ± 3.06/mm^2^) was more than that of axillary nerve fibers (11.33 ± 3.21/mm^2^) without a significant difference. The number of median nerve fibers in Group A (17.67 ± 3.06/mm^2^) was more than that of axillary nerve fibers in Group B (13.00 ± 2.00/mm^2^) without a significant difference. Figure 6 showed different nerve fibers in different groups.

### PCR for MAFBOX and MURF1

**Fig. 7:**
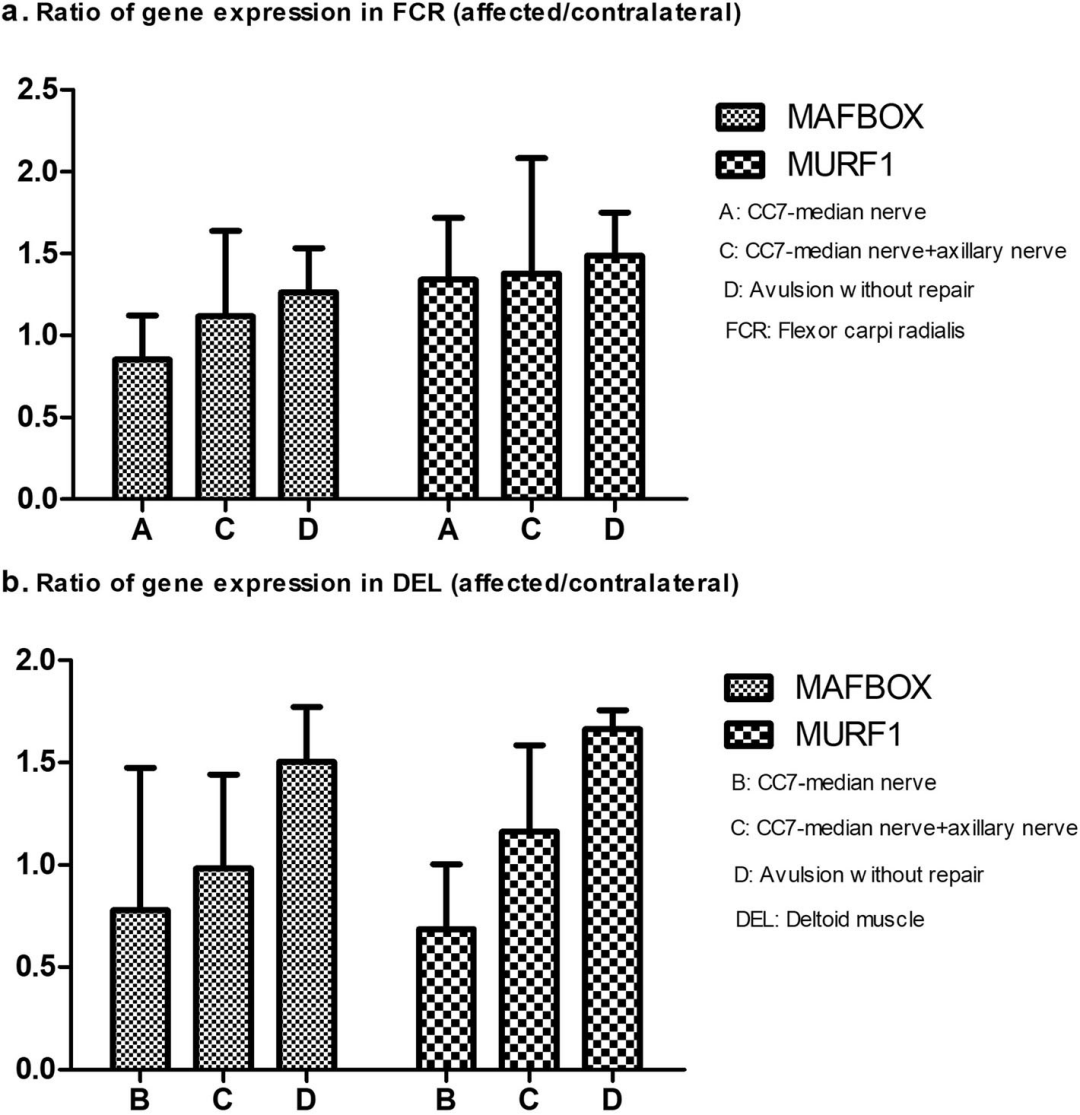
**a** The ratios of gene expressions in FCR (affected/contralateral) in different groups. **b** The ratios of gene expressions in DEL (affected/contralateral) in different groups

By qRT-PCR assay, the ratio of MAFBOX expression in FCR on affected side to contralateral side successively increased from Group A to C to D, but there were no significant differences of the ratios among the three groups. The trend of MURF1 ratios in FCR from Group A to C to D was the same with that of MAFBOX ratios in FCR. (Fig. 7a).

As for DEL, the ratios of MAFBOX and MURF1 rose from Group B to C to D. The ratios in the three groups had no statistical differences among them, no matter for MAFBOX or MURF1. (Fig. 7b).

## Discussion

Brachial plexus injury is one of the severe injuries to the upper limb. It was reported that 10 to 20% of peripheral nerve injuries were brachial plexus injuries [16]. Total brachial plexus root avulsion is difficult to repair. At present, nerve transfer is a primary method for total brachial plexus root avulsion. It is widely accepted that the extraplexal nerve transfer is a feasible way for functional restoration [19, 22]. Extraplexal nerve transfers mainly include intercostal nerve transfer [21], spinal accessory nerve transfer [1], phrenic nerve transfer [10] and cC7 transfer [11]. In patients with total brachial plexus root avulsion, cC7 transfer is usually used to repair median nerve. Clinically, repairing two recipient nerves simultaneously by cC7 transfer has been reported to achieve a certain curative effect [5]. Although spinal accessory nerve is often transferred to the suprascapular nerve for shoulder abduction reconstruction [14], the recovery of shoulder abduction is not satisfying [8]. Recent studies showed that repairing both axillary and suprascapular nerves could achieve effective shoulder abduction and external rotation [12]. This study was designed to explore the efficacy of cC7 transfer to median and axillary nerves simultaneously.

In the study, all the experimental rats were healthy before surgery. No injury occurred on either side before surgery. For the identity of each group of experiments, we have specified the right side of each rat was selected as the injure side in all groups. In Group C, two rats died after operations. One of them died due to overdose of anesthetics. The other rat died because of the excessive bleeding due to vascular injury. In the study, behavioral tests, EMG examination, mean muscle fiber cross-sectional area, nerve fiber count and gene expression related to muscle atrophy were used to demonstrate the efficacy of cC7 transfer. CMAP and the number of nerve fibers represented nerve regeneration after nerve repair. In Group D, the rats were not repaired with total brachial plexus avulsion, so there was no emergence of compound muscle action potential (CMAP) in all peripheral nerves of the right upper extremity in the group. The cross-sectional area of muscle fiber reflected the degree of muscle atrophy and regeneration. Based on previous studies, the expressions of MAFBOX and MURF1 were positively correlated with muscle atrophy [6]. All the results showed there were no significant differences in the efficiency of median nerve recovery between Group A and C. Both of Group A and C improved median nerve regeneration. The above results indicated that cC7 transfer to both median and axillary nerves would not affect the recovery of median nerve, compared with cC7 transfer to median nerve alone. Anatomical reports showed 17,000–40,000 myelinated nerve fibers in cC7 nerve root [4]. The average number of myelinated nerve fibers was about 14800 in the median nerve [20], while it was 2704 in axillary nerve [26]. The number of nerve fibers in cC7 nerve root was more than that of any other recipient nerve. CC7 nerve root had the capacity to provide enough dynamic nerve fibers to both of median and axillary nerves theoretically, which was consistent with our results.

Many studies recommended suprascapular and axillary nerves should be repaired simultaneously to obtain better shoulder joint function, because repairing suprascapular nerve alone was not enough for the reconstruction of shoulder abduction [12, 15]. In aspect of the number of axillary nerve fibers, the results in Group B and C were statistically more than that in Group D. There was no statistical difference in the number of axillary nerve fibers between Group B and C. The results of behavioral test and EMG also showed deltoid recovery in Group B and C. No statistical difference existed between the two groups. All these results above indicated that axillary nerve could be repaired no matter by cC7 transfer to axillary nerve alone or by cC7 transfer to axillary and median nerves.

As for the cross-sectional area of DEL fibers, although there were no statistical differences among Group B, C and D, the mean cross-sectional areas of DEL fibers decreased successively from Group B to C to D. The results demonstrated muscle atrophy was related to denervation. Nerve transfer made nerve regeneration, which could reduce muscle atrophy. There was functional impairment in Group D compared with Group B and C, while no statistical demonstration was showed in muscle fiber count of DEL. Because the completion of an action requires not only sufficient muscle fibers, but also enough neural dynamics. Either the number of axillary nerve fibers in Group B or C was significantly more than that in Group D, which meant either the neural dynamic in Group B or C was more powerful than that in Group D. Another reason might be the time from operation to biopsy, which was not long enough to make muscle completely atrophy in Group D. It induced no statistical differences of the cross-sectional area in DEL among groups.

Bodine SC et al. [2] reported only a small subset of genes was universal in all atrophy models. Two of these genes encoded ubiquitin ligases: Muscle RING Finger 1 (MuRF1) and Muscle Atrophy F-box (MAFbx). Overexpression of MAFbx in myotubes produced atrophy, whereas mice deficient in either MAFbx or MuRF1 were found to be resistant to atrophy. The models were the rats with denervation or immobilization or unweighting. They were a little different from our models. In our study, there were four groups, including one denervation group and three nerve regeneration groups. In our research, the results of MAFbx and MURF1 expression in DEL coincided with the result of the mean cross-sectional areas of DEL, while the data in gene expression in FCR was not consistent with the mean cross-sectional areas of FCR. Muscle regeneration takes time. When the innervation to the muscle occurs, the time course of inducing the change of MAFbx and MURF1 is unknown yet, which is an intriguing question. Maybe the time from operation to biopsy is not long enough to show the different change of MAFbx and MURF1 between innervation and denervation groups. We think the denervation process with the gene regulation might not just be the opposite process of innervation. Whether the innervation process had correlation with these two genes needs a further study.

The recovery proportion of axillary nerve was less than that of median nerve in terms of behavior tests, EMG and the number of nerve fibers in the study. The main reason is the anatomy of axillary nerve, which passes through the quadrilateral foramen. Axillary nerve could be easily compressed in the quadrilateral foramen, especially during the process of nerve growth. The compression will influence nerve fiber growth. In addition, the diameter of axillary nerve is smaller than that of median nerve. Most of cC7 nerve fibers will grow into median nerve.

Previous meta-analysis showed that the average effective rate of cC7 transfer to median nerve was 50%, which was close to our result (70%) [13]. There were some clinical and experimental reports on cC7 transfer to two recipient nerves simultaneously. Gao et al. [7] reported cC7 transfer to median nerve and biceps branch or median nerve and triceps branch. The recovery rates of motor function were 68.2, 66.7 and 20% in median nerve, biceps branch and triceps branch, respectively, which indicated that cC7 transfer to two recipient nerves simultaneously could achieve effective results. Pan et al. reported an animal experiment on cC7 transfer to median and musculocutaneous nerves. The target muscles innervated by median and musculocutaneous nerves had regeneration [18], but the details about the recovery rate of muscle strength were not reported. Chuang et al. [5] also found that cC7 could be used to repair median and musculocutaneous nerves simultaneously. The recovery rates of finger and elbow flexion were 39 and 82.6%, respectively.

Terzis et al. [23] used hemi-cC7 nerve root to repair axillary nerve. 20% patients achieved M3 or above in DEL. In our experiment research, the axillary nerve was repaired with total cC7 nerve root. The effective rate was 60% in rats, which was quite different from Terzis’ report. There were two probable reasons. One was the difference of nerve growth speed between human beings and rats. The other was the difference of donor nerve. Terzis et al. used hemi-cC7 nerve root as the donor nerve, while we adopted total cC7 nerve root for transfer. There was no animal experimental report on cC7 transfer to both of median and axillary nerves before.

There were some limitations in this study. First, there was a lack of a more accurate behavior test for shoulder abduction and external rotation. Secondly, the muscle tension measurement was an important method for evaluating muscle recovery, while we did not use it in our experiment. Third, the number of samples in the research is small, which made the results not so convincing. Afterwards, we’ll increase the number of samples in the future research. In the behavioral tests and EMG results, we compared the percentages of effectiveness among groups. Due to the small number of samples, the statistical analysis seemed to lower the power of the test. The amplitude and latency of CMAP were not assessed. In the future study, we’ll use amplitude and latency as indicators for nerve regeneration evaluation. In addition, there was a remodeling process of cortical plasticity after cC7 transfer, which would be the focus in the future research.

## Conclusions

Compared with cC7 transfer to median nerve, cC7 transfer to both median and axillary nerves did not affect median nerve recovery. The deltoid muscle could also be restored. In this animal experimental research, the recovery proportion of axillary nerve was less than that of median nerve.

## Data Availability

The datasets used and analysed during the current study are available from the corresponding author on reasonable request.

## Abbreviations

ANOVA: Analysis of variance
cC7: Contralateral cervical 7 nerve
CMAP: Compound muscle action potential
DEL: Deltoid
EMG: Electromyogram
FCR: Flexor Carpi Radialis
HE: Hematoxylin and eosin
MAFbx: Muscle Atrophy F-box
MuRF1: Muscle RING Finger 1
S-D: Sprague-Dawley
SPSS: Statistical Package for Social Sciences
TBPA: Total brachial plexus avulsion
